# Benzodiazepine agonist treatment for patients with benzodiazepine dependence undergoing opioid agonist treatment: a study protocol for the randomized controlled trial BMX-BAR

**DOI:** 10.1186/s13063-024-08692-8

**Published:** 2025-01-02

**Authors:** Fatemeh Chalabianloo, Lars Thore Fadnes, Jörg Assmus, Jon Mordal, Kristin K. Solli, Kjetil S. Dale, Christina D. Andersen, Silvia Zavenova, Beathe H. Rønning, Andreas W. Blomkvist, Martin Ryssdal, Wasifa S. J. Butt, Anne Marciuch, Anne G. Ørmen, Christian Ohldieck, Else-Marie Løberg, Kjell Arne Johansson, for the BMX-BAR and NORCATS study group, Fatemeh Chalabianloo, Lars Thore Fadnes, Jörg Assmus, Jon Mordal, Kristin K. Solli, Kjetil S. Dale, Christina D. Andersen, Silvia Zavenova, Beathe H. Rønning, Andreas W. Blomkvist, Martin Ryssdal, Wasifa S. J. Butt, Anne Marciuch, Anne G. Ørmen, Christian Ohldieck, Else-Marie Løberg, Kjell Arne Johansson, Svanhild Mellingen, Maria K. Olsvold, Mette H. Nordbotn, Beate H. Trettenes, Tine U. Berger, Kristin Sannerud, Christine Sundal, Marianne Pierron, Henriette Moe, Zainab Alibhai, Karoline N. Helgøy, Susanne Vedaa, Line Holtan, Heidi F. Kristiansen, Britt K. Haugen, Jeanine T.H. Karlsen, Richard Kaspersen, Linn C.W. Digranes

**Affiliations:** 1 Department of Addiction Medicine, Haukeland University Hospital, Bergen, Norway; 2 Department of Global Public Health and Primary Care, University of Bergen, Bergen, Norway; 3 Center for Clinical Research, Haukeland University Hospital, Bergen, Norway; 4 Department of Addiction Medicine, Vestfold Hospital Trust, Tønsberg, Norway; 5 Norwegian Center for Addiction Research, University of Oslo, Oslo, Norway; 6 Department of R&D, in Psychiatric Health Care, Akershus University Hospital, Oslo, Norway; 7 Department of Addiction Medicine, Telemark Hospital Trust, Skien, Norway; 8 Department of Addiction, University Hospital of North Norway, Tromsø, Norway; 9 Institute of Clinical Medicine, University of Tromsø, The Arctic University of Norway, Tromsø, Norway; 10 Department of Addiction, Akershus University Hospital, Lørenskog, Norway; 11 Department of Addiction, Østfold Hospital Trust, Grålum, Norway; 12 Division of Psychiatry, Haukeland University Hospital, Bergen, Norway; 13 Institute of Clinical Psychology, University of Bergen, Bergen, Norway

**Keywords:** Benzodiazepines, Opioid agonist treatment, Polydrug use, Randomized controlled trial

## Abstract

**Background:**

There is a lack of knowledge on effective treatment methods for comorbid benzodiazepine dependence in populations undergoing opioid agonist treatment (OAT). Tapering and discontinuation of benzodiazepines has long been considered the standard treatment, even though there is limited evidence for this practice. There is also limited research on benzodiazepine agonist treatment; however, peer and clinical experiences indicate that such approaches may be beneficial for a subgroup of the patients with long-lasting benzodiazepine dependence not responding to other treatment approaches. A randomized controlled trial will be conducted to compare the efficacy and safety of stabilizing agonist treatment using prescribed benzodiazepines with standard treatment in reducing illicit benzodiazepine use.

**Methods:**

The target sample is 108 participants at outpatient OAT clinics in six Norwegian cities/counties (Bergen/Vestland, Tønsberg/Vestfold, Skien/Telemark, Fredrikstad/Østfold, Tromsø/Troms, and Lillestrøm/Akershus). The main inclusion criteria are benzodiazepine dependence of ≥ 5 years, using ≥ 5 days a week during the last month, and previous attempts at tapering. Participants will be randomly assigned to receive either a 26-week benzodiazepine stabilizing treatment (15–30 mg diazepam or 50–100 mg oxazepam daily), or a 20-week tapering using the same medications and equivalent initial dosages. All participants will be given access to consultations from OAT therapists with psychosocial follow-up in accordance with current clinical practice.

The primary outcome is the use of illicit benzodiazepines assessed by observed urinary tests at week 24. Secondary outcomes include mental health symptoms, quality of life, cognitive performance, violence risk, other substance use, treatment retention, and life satisfaction. Additionally, the study will assess treatment-related adverse events as well as the cost-effectiveness of the intervention.

**Discussion:**

This is the first randomized controlled trial of benzodiazepine agonist treatment for benzodiazepine dependence. The research project will assess efficacy and safety of stabilizing treatment with prescribed benzodiazepines compared to benzodiazepine tapering and discontinuation regarding use of illicit benzodiazepines and accordingly well-being of patients with concurrent benzodiazepine and opioid dependence undergoing OAT. If the intervention is found to be efficacious and safe, it will be considered one of the options to standard treatment for this patient group.

**Trial registration:**

EU trial number: EudraCT: 2021–004981-37. Registered on December 13, 2021.

## Introduction

### Background and rationale {6a}

Use of benzodiazepines is common among people with substance use disorders (SUD) [1–3], with a high prevalence ranging between 61% and 94% reported in patients with opioid use disorders [4]. Historically, benzodiazepine dependence is recommended to be managed with short-term tapering regimens combined with non-pharmacological and psychological interventions [4, 5]. The effect and safety of short-term detoxification including benzodiazepine tapering in patients with SUD is uncertain [6, 7]. Tapering interventions and complete abstinence-based strategies have often not been successful, especially among those with severe and long-term dependence [6]. Short-term detoxification is even attributed to an increased risk of life-threatening complications such as seizures and psychotic reactions [8]. Although substitution treatment is not recommended, a large proportion of benzodiazepine-dependent patients have continuously been prescribed benzodiazepines, mainly by primary care physicians [9]. Recent research has also confirmed such prescriptions among 30–50% of the Norwegian patients undergoing opioid agonist therapy (OAT) [1]. The Norwegian OAT guidelines recommend gradual tapering and discontinuation of benzodiazepines as the main recommendation, but also propose to consider benzodiazepine stabilizing agonist treatment for patients with severe and long-lasting dependence who have not succeeded in tapering [10]. It is acknowledged that there is limited research supporting this recommendation, although user-reported and clinical experiences indicate that such approaches may be beneficial in improving the quality of life for some patients with long-term and regular use of benzodiazepines.

Reports have shown that concurrent dependency to benzodiazepines and other addictive substances in patients undergoing OAT complicates the treatment course and reduces the chance to improve health and quality of life [1, 11]. These patients demonstrate more frequently symptoms of mental illness, use multiple substances, and have more impaired psychosocial functioning compared to patients without benzodiazepine dependence [12]. It is uncertain whether these findings are due to differences between patient groups (e.g., in the extent of mental illness) and/or use of benzodiazepines per se. Clinical experiences indicate that the majority relapses and continues to use benzodiazepines acquired illicitly despite tapering attempts [4, 12, 13]. There is a lack of evidence on the effect and safety of standard treatment approaches as well as benzodiazepine substitution treatment in populations with severe SUD including those undergoing OAT [5, 14]. In a small, non-randomized controlled study on patients undergoing methadone maintenance treatment (total *n* = 66; 33 in each group), the proportion using illegally acquired benzodiazepines was lower in patients receiving benzodiazepine substitution treatment compared with patients who tapered these agents and discontinued (77% vs. 27%, and 65% vs. 14%) after 2 and 12 months, respectively [15]**.** Thus, there is an urgent need to conduct randomized controlled trials on the benefit and risks of benzodiazepine stabilizing agonist treatment for patients with benzodiazepine dependence undergoing OAT as compared to standard treatment approaches.

Use of benzodiazepines may in turn be related to several clinical characteristics and symptoms. Generally, it is associated with impaired cognitive functioning, in addition to an increased risk of violent behavior [16]. Some register-based studies have shown that patients undergoing OAT who were prescribed benzodiazepines had an increased risk of overdose death compared with those without such prescriptions [17, 18]. In a Norwegian study, clonazepam was often recognized as a contributing agent in emergency units for drug intoxications [7]. In another study, it was frequently found in addition to opioids in toxicological analyses related to overdose death [19]. This is contrary to the Norwegian prescribing pattern in 2014–2015 where the prescription rate of both diazepam and oxazepam (less potent than clonazepam) were 50% higher than of clonazepam. Police confiscations clearly confirm the distribution of the most common illicitly acquired benzodiazepines (among others clonazepam and alprazolam, but not diazepam or oxazepam) in drug-related intoxications [7, 19]. Additionally, contaminated drug supplies have recently been an increasing and worrying issue in Norway and some other European countries where potent synthetic opioids such as nitazenes have been detected in substances sold as other opioids or benzodiazepines [20]. These findings support that the increased risk is related to use of more potent and illicitly acquired and in some cases contaminated benzodiazepines such as clonazepam and alprazolam rather than controlled use of prescription ones such as diazepam and oxazepam which usually are less potent and seldom used illicitly in Norway. A large cohort study, on the other hand, among 353,576 patients receiving stable long-term treatment with benzodiazepines showed that discontinuation was associated with small absolute increases in mortality and other potential harms, including nonfatal overdose, suicide attempt, suicidal ideation, and emergency department visits [21]. These results suggest benzodiazepine discontinuation among patients prescribed for stable long-term treatment may be associated with unanticipated harms, and that efforts to promote discontinuation should carefully consider the potential risks of discontinuation relative to continuation.

Despite the high disease burden and related risks among people with benzodiazepine dependence, evidence on effective treatment methods is lacking. We will conduct a multi-center randomized controlled trial on patients undergoing OAT with long-lasting and hard-to-treat concurrent benzodiazepine dependence to study the efficacy and safety of benzodiazepine agonist treatment.

### Objectives {7}

The overall objective of the trial is to investigate the effect of stabilizing agonist treatment with prescribed benzodiazepines (diazepam or oxazepam) in reducing the use of illicitly acquired potent benzodiazepines (clonazepam and alprazolam) among people on OAT with benzodiazepine dependence compared to tapering. The central hypothesis is that stabilizing treatment with less potent prescribed benzodiazepines such as diazepam and oxazepam in patients with severe and long-lasting benzodiazepine dependence will result in significant reduction in use of illicit and more potent benzodiazepines such as clonazepam and alprazolam. Accordingly, this may reduce risk and improve health and quality of life in this population.

The primary objective of the study is efficacy of stabilizing agonist treatment with daily doses of 15–30 mg diazepam or equipotent doses of 50–100 mg oxazepam in reducing illicitly acquired potent benzodiazepines use measured at 24 weeks intervention. Secondary objectives are to compare mental health symptoms, cognitive functioning, and health-related quality of life between intervention and control groups, as well as to study differences in violence risk, overdoses, and adverse events. Other secondary objectives are to examine differences in treatment retention and satisfaction, and use of alcohol and other substances between the groups.

### Trial design {8}

This project is a multi-center, randomized controlled, open and flexible dose trial comparing a 26-week stabilizing agonist treatment by using diazepam (15–30 mg a day) or oxazepam (50–100 mg a day) with a 20-week gradual tapering using the same medications and equivalent initial doses.

## Methods: participants, interventions, and outcomes

### Study setting {9}

This study will be conducted in outpatient OAT clinics in six Norwegian cities/counties (Bergen/Vestland, Tønsberg/Vestfold, Skien/Telemark, Fredrikstad/Østfold, Tromsø/Troms and Lillestrøm/Akershus). In total, 108 patients will be recruited. Potential participants will be prescreened in person by a research nurse or OAT staff, and if potentially eligible invited to attend to a formal screening, i.e., eligibility assessment by a physician in line with current clinical practice and trial protocol.

The Department of Addiction Medicine, Haukeland University Hospital (Vestland county, city of Bergen, Norway) is responsible for the treatment and follow-up of more than 1000 patients with opioid dependence receiving OAT, of which almost 35% receive methadone while the remaining mainly receive buprenorphine preparations, seldomly oral morphine depot. All medical interventions are integrated with psychosocial care provided in multidisciplinary outpatient clinics as part of specialist health care system. The outpatient OAT clinics are staffed by medical consultants specialized in addiction medicine in addition to nurses, social workers, and psychologists. Dependent on the overall functioning level and decisional capacity, the follow-up of the patients ranges from daily observed medication and consultations to weekly take-home doses. All the clinical measurements and laboratory data are recorded in the hospital journal system. A similar OAT model and organization is also applied in the other involved South-East and North region counties as part of specialist health care system in Norway. These centers include approximately 2000 OAT patients in total with similar clinical and sociodemographic characteristics. The applied OAT platform allows frequent and close clinical observations and follow-up of the participants to increase safety and to ensure a high quality of data collected.

### Eligibility criteria {10}

The study population is treatment-seeking adults undergoing OAT who fulfil all the inclusion criteria and do not have any of the exclusion criteria.

Inclusion criteria are:

- Benzodiazepine dependence according to International Statistical Classification of Diseases and Related Health Problems 10th Revision (ICD-10) under the following conditions:
  - Minimum duration of ≥ 5 recent years (as a cut-off for severity of dependence)
  - Self-reported use of ≥ 5 days a week during last month
  - Minimum dose used daily is equivalent to ≥ 15 mg diazepam
- Previous at least one failed attempt at outpatient or inpatient tapering of benzodiazepines
- Capable of giving signed informed consent, which includes compliance with the requirements and restrictions listed in the informed consent form and in the study protocol.

Use of illicitly acquired potent benzodiazepines (clonazepam and/or alprazolam) will be verified by a high specific urine drug screening test (UPLC MS–MS) at baseline. Failure to respond to previous treatment is defined as a relapse to dependent use during tapering or after completing treatment.

Exclusion criteria are:

- Severe respiratory failure (Global initiative for chronic obstructive pulmonary disease “GOLD” grade 3–4)
- High risk of violent behavior (current violence episodes, i.e., during the last 6 months)
- High risk of substance related overdose (current overdoses, i.e., during the last 3 months)
- Severe cognitive impairment (IQ < 70; assessed if needed based on clinical decision)
- Severe psychosis (current psychotic symptoms and functioning, i.e., during the last 6 months based on clinical decision)
- Severe depression and high suicide risk (current episodes, i.e., during the last 6 months based on clinical decision)
- Patients who are already being stabilized with continuously prescribed benzodiazepines (including diazepam or oxazepam during the last 4 weeks prior to baseline assessment)
- Pregnancy and breastfeeding (female participants should use a safe method of contraception; in doubtful cases, a negative pregnancy test will be required)
- Challenges related to ability to understand, consent, or willingness to collaborate in following up of the study protocol.

The risk of possible drug interactions will be considered individually at the time of eligibility assessments, which in some cases may result in exclusion from participation. Combination of benzodiazepines with opioids, alcohol, and other central nervous system depressants may cause respiratory depression and increase the risk of overdose. Otherwise, there are few known clinical important interactions between benzodiazepines and other medications.

### Who will take informed consent? {26a}

Participants will receive comprehensive information about the study during recruitment visits to ensure informed consent and assent. Trained research staff will obtain written informed consent from the patients who wish to attend.

### Additional consent provisions for collection and use of participant data and biological specimens {26b}

Participants will be asked if they are willing to participate in an ancillary qualitative study investigating self-perceived effects of the interventions. If so, an additional written informed consent will be obtained.

## Interventions

### Explanation for the choice of comparators {6b}

The comparator is tapering with diazepam or oxazepam in maximum 20 weeks according to the applied clinical procedure in OAT clinics which is based on the Norwegian OAT guidelines recommending gradual tapering and discontinuation of benzodiazepines as the standard treatment of benzodiazepine dependence.

### Intervention description {11a}

Participants in the intervention arm will receive stabilizing agonist treatment with 15–30 mg/day diazepam or equivalent dosages of 50–100 mg/day oxazepam in 26 weeks. Participants in the standard arm will receive tapering with diazepam or oxazepam in maximum 20 weeks according to the applied clinical procedure in OAT clinics. The type of benzodiazepine prescribed (diazepam or oxazepam), the start dosages, and the duration of tapering will be based on the degree of dependence, the dosages of illicit benzodiazepines used prior to study entrance, and the individual’s clinical condition [22]. Accordingly, a customized tapering plan will be suggested to each participant within the framework of the study procedures.

For both arms, treatment initiation and follow-up will be conducted at OAT outpatient clinic where they already receive OAT and relevant care including voluntary psychosocial interventions. The study medications will be prescribed by the physicians in the OAT clinics in accordance with the protocol and will be delivered either at the OAT clinic or at a pharmacy in line with the applied standards for OAT follow-up. Commercial tablets and standard medication labels will be used, tagged with trial log numbers. All the costs related to the medications, preparations, and observed intakes will be covered by the OAT clinics (through public assigned funds). The medications will be used in line with national guidelines, and the project will strive to comply with the Good Clinical Practice (GCP) [10, 23].

### Criteria for discontinuing or modifying allocated interventions {11b}

The stop criteria for the individual participant are defined at least based on non-compliance, unexpected adverse events, or other safety considerations such as use of large amounts of highly potent street benzodiazepines with clinically observed signs of overdosing. In addition, medication can be withheld in the case of deviation from urinary test procedures that can affect measuring the trial primary outcome. Participants who have discontinued protocol-based treatment will be motivated to continue to participate in all remaining research interviews and assessments. For those participants who revoke their consent for the entire study, no further data will be collected from the participant.

### Strategies to improve adherence to interventions {11c}

Medication choice between oxazepam and diazepam will be individualized based on patient preferences, medical history, and present health condition. The prescription method will also be assessed individually and based on the patient’s treatment course (i.e., prescribed by the clinic physician and ordered through the related clinic or pharmacy). All participants will be encouraged to initiate the treatment according to the protocol, with data collected on prescription pickup and treatment initiation dates. In addition, for those receiving medications in the clinics, frequencies, and observation of doses taken will be noted. Self-reported data on medication adherence and compliance will be obtained for all participants. Medication adherence and compliance will be assessed through a combination of prescription pickup frequency, self-reported adherence, observed intake, and urinary tests.

Related authorized clinical study-monitoring organs in each health county will visit the study sites on a regular basis to ensure the following requirements: informed consent process, reporting of adverse events and all other safety data, adherence to protocol, maintenance of required regulatory documents, facilities, and data completion on the case report files (CRFs) including source data verification. Additionally, a data monitoring committee (DMC) comprising two independent professionals (a clinician and a researcher) and a statistician will ensure the safety and wellbeing of trial patients and will assist and advice the coordinating and principal investigators to protect the validity and credibility of the trial.

### Relevant concomitant care permitted or prohibited during the trial {11d}

All the participants will receive OAT and the related care as usual. Study medications will mainly be administered under supervision by the OAT clinic or pharmacy staff. Individually assessed, the participants will receive some of the doses as take-home-dosages for self-administration. Assessments of observed intake frequency and “take-home doses” will follow the same agreement that applies to OAT medications, and any changes will be assessed by the prescribing OAT physician after discussion in interdisciplinary team. Project managers will be informed of any changes. In any case, all participants should attend the OAT outpatient clinic daily or at least once weekly for clinical observations. Every participant should, regardless of the delivery agreement, have frequently clinical observations and assessments for the first 2 weeks after starting prescription (in both study arms) to ensure treatment safety. After this period, they follow the delivery agreement as before.

**Fig. 1:**
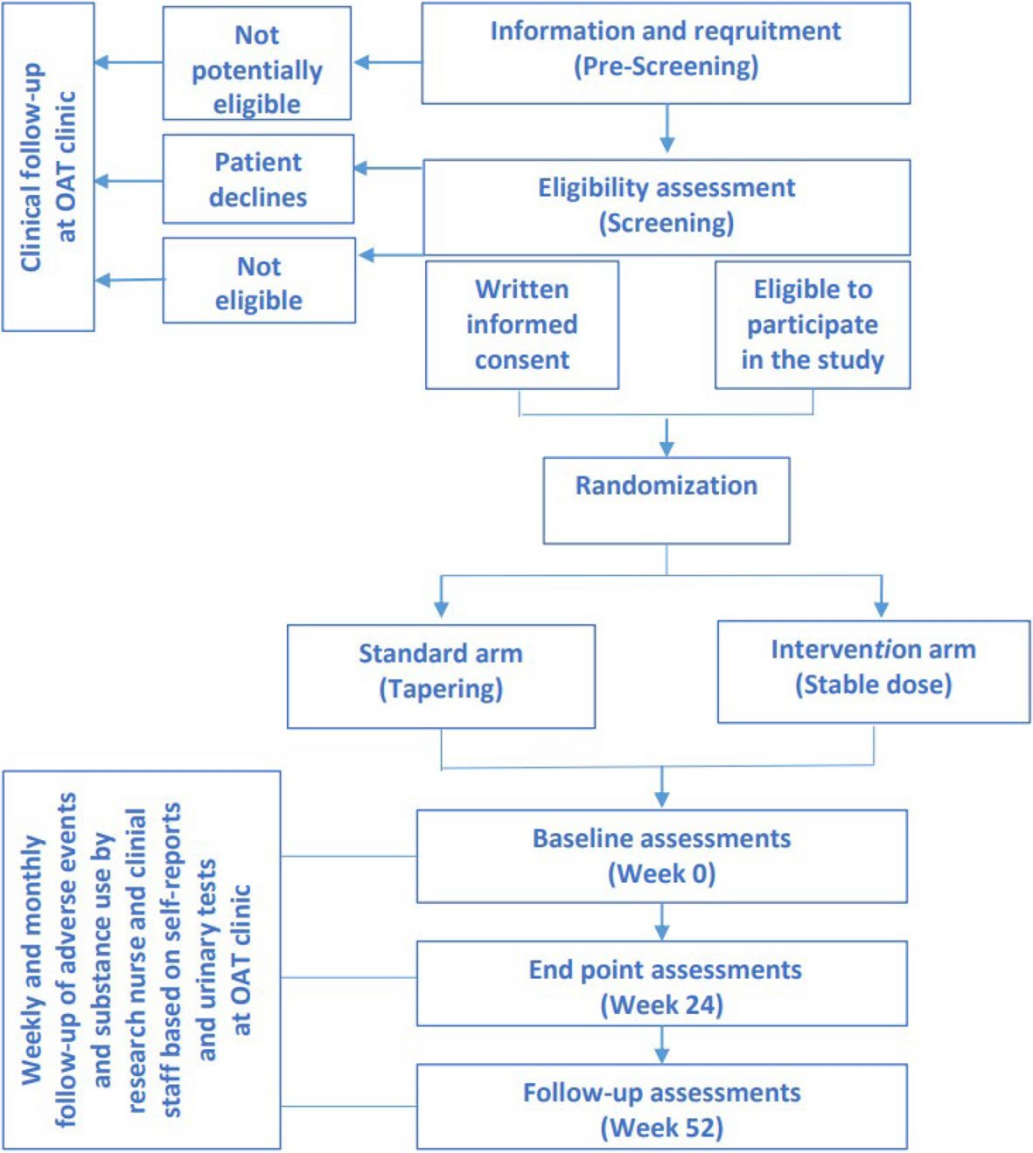
Flow-chart of the study procedures. Legend: Potential participants will be screened for eligibility. Individuals, who meet the eligibility criteria and provide written informed consent to participate, will be randomized either to a stabilizing dose with diazepam or oxazepam, or tapering using the same medications. The primary endpoint is at week 24 with a follow-up visit at week 52. OAT: opioid agonist treatment

Clinical and biological follow-up of participants and systematic report of potential adverse effects will be organized according to international GCP guidelines. The participants will be assigned to the scheduled assessments by research nurses and/or clinic physicians in line with the study protocol. The assessments will be performed at baseline, and at week 24 in addition to a follow-up assessment at week 52 after entering the trial. The OAT staff or research nurses will follow up the participants weekly at OAT clinics or by home-based visits if needed, with consultations including self-reports on illicit use of benzodiazepines and other substances, adverse events as well as monthly randomized urine drug screening tests during the trial period (Fig. 1 and Table 1).

**Table 1:** Schedule of activities during the study period

	Pre-screening and screening by research nurse/physician	Assessment at Baseline by research nurse/physician	Assessment at week 24 by research nurse/physician	Assessment at week 52 by research nurse/physician	Weekly follow-up by research nurse/clinic staff
Eligibility criteria	X				
Written Informed consent	X				
Randomization	X				
Observed urinary tests		X	X	X	X (monthly)
Self-reported drug use		X	X	X	X
SCL-10		X	X	X	
EQ-5D-5L		X	X	X	
BVC		X	X	X	
Reaction time		X	X	X	
Treatment satisfaction		X	X	X	
Days out of treatment		X	X	X	X
Recorded overdose		X	X	X	X
Adverse effects			X		X

*SCL-10* Hopkins symptom checklist, *EQ-5D-5L* Euro Quality of life, *5-dimensional *5-level questionnaire, *BVC* Brøset violence checklist

### Provisions for post-trial care {30}

At the end of trial period, each participant will be individually assessed to receive further clinical follow-up as indicated. Participants who receive stabilizing treatment with prescription benzodiazepines during the 26-week study period will undergo individual clinical assessments upon trial completion. Some participants may continue with stabilizing treatment, while others will have their prescriptions stepped down and discontinued following current guidelines. Participants in the standard treatment arm will receive ongoing treatment and follow-up using conventional approaches after tapering of medications.

Participants will be informed about these individual assessments at the study enterance. Clinical observations throughout the study period will provide insights into outcomes for participants in each study arm. Decision-making following the project’s conclusion will be based on these outcomes, aligning with the conditions governing benzodiazepine continuation outlined in the study protocol. Assessments will occur both upon completion of the intervention (after week 26) and at the end of the project period (after week 52).

### Outcomes {12}

Primary outcome measure is:

- The difference between the groups in the use of illicit benzodiazepines based on supervised urine drug screening tests measured during the 24-week trial.

Secondary outcome measures collected at baseline and week 24 will include:

- Mental health symptoms score using Hopkins symptom checklist (SCL-10) [24]
- Health-related quality of life score using 5-dimensional, 5-level Euro Quality of life questionnaire (EQ-5D-5L) [25]
- Reaction time for cognitive performance using a simple reaction time test [26]
- Risk of violence behavior using Brøset violence checklist (BVC) [27]
- Satisfaction with the treatment using visual analog scale (VAS) from 0 to 10
- Retention rate in OAT (number of drop-out days during the trial period)
- Self-reported frequency of use of alcohol and illicit substances including benzodiazepines, and urine drug screening for alcohol and illicit substances other than benzodiazepines
- Number of non-fatal overdoses and death (if any)
- Cost-effectiveness of intervention

### Participant timeline {13}

The time schedule of enrolment, interventions, assessments, and visits for participants is shown in Table 1.

### Sample size {14}

**Fig. 2:**
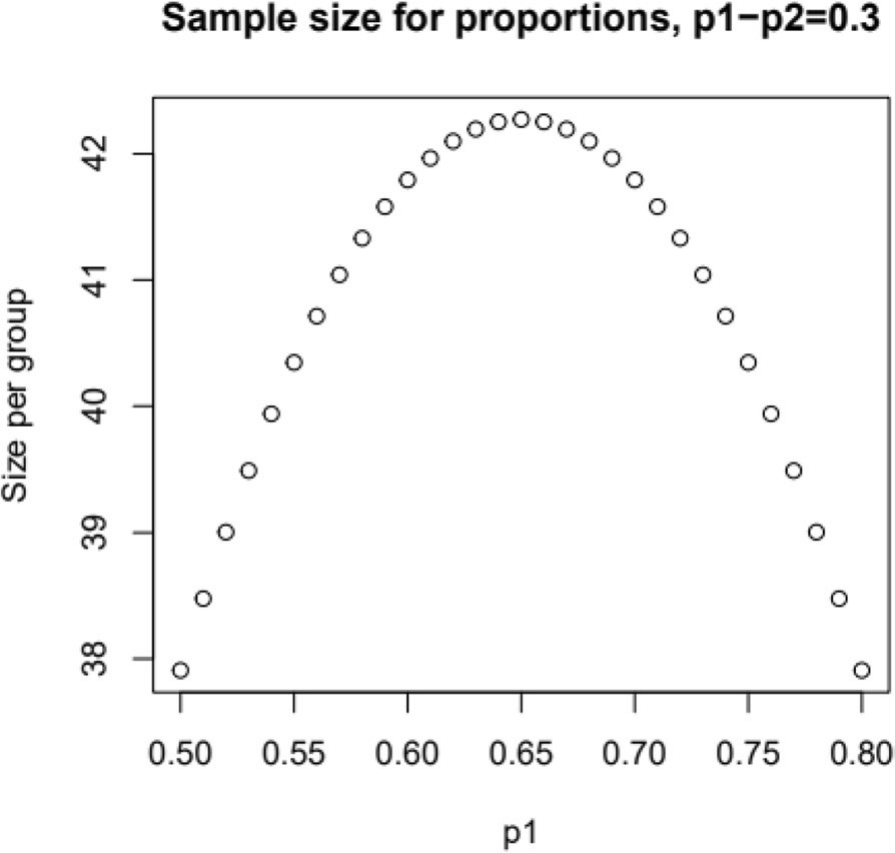
Study sample size

The sample size calculation is based on clinical experiences due to no existing empiric assumption. The prevalence of use of illicit benzodiazepines during standard treatment is assumed to vary from 50% to 80%. We have used an “illegal” index which is defined as the proportion of positive urinary tests confirming the use of illicit benzodiazepines and is considered continuous. We set the minimal clinically relevant difference between the groups—regarding the illegal index at week 24—to 0.3, and the required power to 0.8 at two-sided significance level of 0.05. There is no valid data to estimate the standard deviation. Assuming a risk of 0.5 to 0.8 for the use of illicit benzodiazepines in the standard treatment group (p1), and an expected difference of 0.3 between the two arms (p1-p2), we need 43 patients per group (Fig. 2). Assuming a drop-out rate of 20% we need 54 patients in each group, 108 in total, to achieve sufficient power [28].

### Recruitment {15}

Enrollment was commenced autumn 2022 and will continue until the required number of eligible participants is enrolled in the trial. For both arms, all the clinical stages of the study—including recruitment, information, obtaining written consent, clinical interviews, and completing the study surveys using appropriate instruments, and treatments—will be performed by research nurses and/or physicians to ensure independence. Six OAT sites are involved in the trial conduction to obtain the required sample size.

## Assignment of interventions: allocation

### Sequence generation {16a}

Eligible participants will be randomized digitally with a 1:1 ratio into intervention or standard treatment for inclusion in the trial. A computer-generated blocked, site stratified randomization schedule will be developed by an independent statistician and uploaded to the study database.

### Concealment mechanism {16b}

Randomization will be performed by research nurse or the trial site investigator within the study database and technically secured against manipulation in the database.

### Implementation {16c}

The allocation sequence will be generated by an independent statistician. The research nurses or physicians will enrol participants in the study and assign them to the interventions. An independent statistician not involved in providing the clinical data will analyze the data collected.

## Assignment of interventions: blinding

### Who will be blinded {17a}

Complete blinding is not considered feasible although some masking measures such as blinding of analysts of the data will be taken (blinding for the trial arms for the analysts). Randomization will be disclosed to the researchers, participants, and clinical staff providing treatment and follow-up. Patients will be informed of key elements in the delivery of the respective intervention or standard treatment and follow-up they are randomized to, but not on the hypotheses for the study.

### Procedure for unblinding if needed {17b}

The trial is not blinded for the participants, researchers, or care givers. However, the trial statistician will be blinded to the allocation and unblinded after data analysis is completed.

## Data collection and management

### Plans for assessment and collection of outcomes {18a}

To ensure correct adherence to standard operating procedures (SOPs), all system users will be trained and evaluated on a regular basis. The research nurses will collect the trial data at baseline and at trial week 24, in addition to weekly self-reported substance use, adverse events, and other research-related data, as well as the randomized monthly urinary screen tests according to Table 1.

The urine drug screening tests as the measure of primary outcome will be taken at the OAT clinics or other planned units under the observation of the staff. A high specific laboratory analysis method (UPLC MS–MS) will be used to differentiate between the benzodiazepine types identified in the urinary samples (those illicitly acquired, i.e., clonazepam and alprazolam, and the prescribed ones, i.e., diazepam and oxazepam). The samples will be taken randomly in a chosen week per a 4-week period. Randomization will be programmed by an independent researcher and is aimed to reduce the effect of any variations in the intake of illicit benzodiazepines between the intervention and the standard arm in relation to the time of sampling. The patients will be informed Monday of the week that they need to provide an observed urinary test before Friday the given week. In case of no show for a urine sample, the patient will receive a warning to take it the subsequent week (up to once). In the event of a missing urine sample or two delays in a month, study medication will be stopped.

Other outcomes will be assessed using standard or semi-structured questionnaires based on Table 1. The study assessments will be completed for each participant at scheduled visits during the study.

Secondary endpoints assessed are:

#### Treatment initiation and medication adherence

Treatment initiation will be assessed as the proportion taking at least one tablet while medication adherence will assess the proportion taking their medicines at least 6 days per week (> 80%) throughout the entire treatment period. Both outcomes will be assessed and reported as a combination of self-reported adherence and observed intake.

#### Weekly self-reported substance use

The weekly self-reported substance use will be measured each week and at week 24. This includes any use and frequency of use (days a week) of illicitly acquired opioids, cannabinoids, and benzodiazepines, as well as alcohol. For benzodiazepines, more detailed information on the type and quantity (mg) of the benzodiazepines used illicitly during the last week will be collected in addition to the information on the frequency of use (days a week).

#### Mental health symptoms

Mental health symptoms will be assessed using Hopkins symptom checklist score (SCL-10) at baseline, and at week 24. The SCL-10 is a structured self-administrated instrument to measure symptoms of mental health disorders and psychological distress. Scores range from 1 (not bothered at all) to 4 (extremely bothered) for each item. To derive the mean item score, the scores will be summed up and divided by the number of items. A mean score of 1.85 or higher will indicate the presence of symptoms of mental and psychological disorders. The mean score of the records at week 24 will be compared between the two study arms.

#### Health Related Quality of Life (HRQoL)

HRQoL will be assessed using EQ-5D-5L at baseline and week 24. The instrument describes and values HRQoL, consisting of the EQ visual analog scale and the EQ-5D descriptive system. The EQ VAS records the patient’s self-reported health on a vertical scale ranging from 0 “Worst Health You Can Imagine” to 100 “Best Health You Can Imagine,” reflecting the patient’s own opinion about the quality of their health condition. The Descriptive System includes five dimensions (5D): mobility, self-care, usual activities, pain/discomfort, and anxiety/depression. Each dimension has five levels (5L): No problems, minor problems, moderate problems, serious problems, and extreme problems. Patients will indicate their health status by selecting the most appropriate statement in each dimension, resulting in a 5-digit number that describes their health status. The mean HRQoL score will be converted to a utility index (ranging from 0 to 1, where 1 is optimal and 0 is worst) using population-based weightings, which is standard in generic HQoL methods. Data at week 24 will be compared between the two study arms.

#### Risk of violence

This secondary outcome will be assessed using the BVC at baseline and week 24. BVC is a 6-item checklist designed to predict imminent violent behavior within a 24-h perspective. The 6 items that reflect the mood setting are confused, irritable, boisterous, physical threats, verbal threats, and attacking objects. Each item is scored as 0 for the absence of behavior and 1 for the presence of behavior, with a maximum total score of 6. The mean score of the records at week 24 will be compared between the two study arms.

#### Reaction time (cognitive performance)

The cognitive performance will be based on the reaction time test (measured 3 consequent times according to the time used to react in milliseconds) at week 24, and the mean score of the records will be compared between the two study arms.

#### Satisfaction with treatment

Satisfaction with the treatment will be compared between the two study arms at week 24 after initiation of treatment using VAS from 0 to 100 where 0 means no satisfaction and 100 means very satisfied. The mean score of the records at week 24 will be compared between the two study arms.

### Plans to promote participant retention and complete follow-up {18b}

Study participants will be encouraged to retain and complete study follow-up. They will also receive a limited economic compensation (500 Norwegian Krones) for the use of time and to increase the motivation to complete the data collections, measurements, and assessments. A list of outcome data will be collected for participants who discontinue or deviate from intervention protocols.

### Data management {19}

All the OAT clinics in the participating sites will use electronic CRFs that will be entered online in a study database through the data collection software (Viedoc®). Alternatively, paper-based CRF will be used by the sites, and then the collected data will be entered in the central electronic CRF by coordinating research nurse. The software is accredited by Helse Vest for national health research and clinical integration. A central data manager at Helse Bergen will assist in designing the CRF, train data collectors in use of the CRF, and aid data export.

### Confidentiality {27}

Only authorized study personnel including the principal investigator, research nurses, and coordinating physicians will have access to CRFs and supporting documents. Data capture and storage will be undertaken using computer systems compliant to GCP. The research data will be stored in an encrypted data server for research with access limited for the principal investigators and coordinating research nurse.

### Plans for collection, laboratory evaluation, and storage of biological specimens for genetic or molecular analysis in this trial/future use {33}

Not applicable. Genetic specimens will not be taken in this trial.

## Statistical methods

### Statistical methods for primary and secondary outcomes {20a}

Analysis methods will follow the CONSORT guidelines as far as possible [29]. All tests will be two-sided. Descriptive results and the estimated efficacy will be presented with 95% confidence intervals (CI). Categorical variables will be summarized as percentages and continuous variables as medians with interquartile ranges or means with standard deviations for variables with a Gaussian distribution. Analyses for the primary endpoint will be undertaken on an intention-to-treat (ITT) basis and reported upon as such. All the randomized and eligible participants will be analyzed based on initial group allocation.

The main endpoint is difference in an illegal index which measures the cumulative use of illicit benzodiazepines (as assessed by monthly supervised urinary tests) at the end of 24 weeks. The illegal index for urinary tests is defined as the proportion of positive urine tests on illicit benzodiazepines for a 24-week trial and is considered continuous. The difference between the groups at the primary time point (week 24) will be assessed using *t*-test and ANCOVA. Risk difference with 95% confidence intervals will be reported.

For the analyses of secondary endpoints and based on the type of outcomes, appropriate analysis methods will be used including ANCOVA, *t*-test, and risk difference with confidence intervals. We have two types of secondary outcomes:

#### Continuous outcomes with baseline measurement

These include monthly urinary tests and weekly self-reports on illicit drug and alcohol use, weekly self-reports on illicit benzodiazepine use, SCL-10, EQ-5D-5L, BVC, reaction time, treatment retention, overdose, and treatment satisfaction. These outcomes are analyzed using ANCOVA, i.e., the linear regression model of the outcome at the primary time point (week 24) depending on the randomization group adjusted for the baseline value of the outcome. The mean difference with confidence interval, coefficient with confidence interval, and the *p*-value will be reported.

#### Continuous outcomes without baseline measurement

These include adverse events. These outcomes are analyzed using the *t*-test for differences in the outcome at the primary time point (week 24). The mean difference with confidence interval and the *p*-value will be reported.

Additionally, we will estimate a linear mixed effects model for continuous outcome variables at all time points depending on time, randomization group, and the interaction between time and randomization group, adjusted using an individual random intercept. We will use a simple contrast. For the outcomes appearing to have linear dependence on time we will also use a linear contrast. For all regression models, we will present the regression table, and for the linear mixed model, we will provide the graphics with mean and 95% confidence interval for each follow-up time point. The ITT analyses will be repeated with the per-protocol dataset. There will be conducted per-protocol analyses as part of sensitivity analyses.

### Interim analyses {21b}

An interim analysis for efficacy of the primary endpoint will be done when 50% of planned sample size is assessed at week 24. We will use a group sequential design without futility and the O’Brien-Fleming alpha spending approach. The interim analysis will be performed by the DMC. The DMC will give a recommendation whether the study should be continued or stopped. Even if not in the model, the DMC can recommend stopping the study for futility [30]*.*

### Methods for additional analyses (e.g., subgroup analyses) {20b}

All patients enrolled in the study will be evaluated with respect to safety-related outcomes according to the treatment they receive. Safety analyses will include summaries of the incidence of all adverse events and serious adverse events that are possibly or probably related to the study intervention and occur during the study treatment period or within 30 days of the last dose of study treatment. Safety analysis will be specified by DMC.

Sub-group analyses will be conducted for exploratory purposes, due to their inherently low statistical power. These analyses will focus on the primary endpoint and confirmatory secondary endpoints to assess the consistency of the investigational intervention effect across various subgroups such as age groups and sex. If a subgroup contains fewer than 10% of the total participants, the subgroup categories may be redefined. The analyses will be conducted using a test for heterogeneity and results will be presented on forest plots presenting the estimated study arm difference and 95% confidence intervals. Further details on the statistical analysis will be provided in the statistical analysis plan.

A cost-effectiveness analysis will be conducted. Effectiveness will be measured with quality-adjusted life years (QALYs), building on primary and secondary results combined with a prospective Markov model. Cost data will be collected alongside the clinical trial employing both healthcare and societal perspective. The analysis will follow the current guidelines and will be conducted by a health economist [31].

### Methods in analysis to handle protocol non-adherence and any statistical methods to handle missing data {20c}

Participants who withdraw from the study treatment will not be censored, as treatment discontinuation is likely to be related to allocation. Deaths will be censored at the last outcome measurement. We will also have a sensitivity analysis assuming missing urine samples is similar as last urine sample taken (to manage missed data for the primary outcome measure). Robustness of the primary outcome will be checked with sensitivity analyses considering censoring and adjusting for potential baseline imbalances.

### Plans to give access to the full protocol, participant-leveldata, and statistical code {31c}

There are no plans for granting public access to the full protocol, participant-level dataset, and statistical code. However, this will be considered by the sponsor if indicated.

## Oversight and monitoring

### Composition of the coordinating center and trial steering committee {5d}

The trial will be administrated and coordinated by Bergen Addiction Research Group (BAR) and Norwegian Research Centre for Agonist Treatment of Substance Use Disorders (NORCATS) at Department of Addiction Medicine, Haukeland University Hospital, Vestland, Bergen in collaboration with the other sites at Vestfold, Telemark, Østfold, Troms, and Akershus. The research consortium brings together and profits from expertise on addiction medicine and clinical trial implementation in the interdisciplinary and highly specialized clinics together with research expertise from BAR and NORCATS in The Department of Addiction Medicine at Haukeland University Hospital and University of Bergen, in addition to the user expertise from the user organization ProLAR employed in the department. Then, the project group is multidisciplinary, involving national and local collaborators, including several partners with extensive clinical experience, members with user perspectives, and researchers from related field. The coordination unit will be led by the principal investigator in close cooperation with the project manager and will have ongoing communication as well as regular meetings with the other sites, i.e., monthly.

The Steering committee (SC) will be responsible for the conduct of the study. The coordination unit will report regularly to the SC for updates on trial progress and potential issues. The SC is the trial decision body, for all scientific and administrative aspects. It will send technical reports to the funder, ethical committees, and regulatory bodies particularly the monitoring organs in Helse Bergen, and financial reports to the funder. SC participants will meet on regular conference calls with customized frequencies.

The coordinating and managing unit will oversee the day-to-day conduct of the trial and will meet weekly to review trial conduct. The composition of this unit includes principal investigators, coordinating research nurses, and study physicians. It will centralize all study information and will report to the steering committee which includes sponsor, national coordinating investigator, and the responsible principal investigators. The coordination unit will coordinate analysis and writing of different outcomes from the study. The trial coordination will also rely on other units, which can be considered as SC sub-committees. These are as follows:

- *Clinical care unit* which will oversee elaborating the clinical procedures for the trial. It will also support the local physicians on the treatment protocols. Physicians in the coordination unit and the participating sites and coordinating research nurse will be members of this unit.
- *Study site units* which will oversee day-to-day implementation at each study clinic in collaboration with the coordination unit. The clinic leaders will generally be involved in the site units.

### Composition of the data monitoring committee, its role and reporting structure {21a}

The main aim of DMC is to ensure the safety and wellbeing of trial patients and to assist and advise the coordinating investigator, steering committee, and the principal investigators, so as to protect the validity and credibility of the trial. The DMC will be comprising two independent clinicians and researchers and a statistician. An agreed DMC charter will describe the roles and responsibilities of the committee, including the timing of meetings, methods of providing information to and from the DMC, frequency and format of meetings, statistical issues, and relationships with other committees. The charter will be in place before the first patient is included.

### Adverse event reporting and harms {22}

Potential adverse events among participants will be managed according to the treatment guidelines [10]. A low rate of adverse effects and toxicity is expected with the applied benzodiazepines. Complete lists on the reported adverse effects are described in the summaries of product characteristics for oxazepam and diazepam [32, 33] as the reference safety information. All serious adverse events (SAE) reported to the sponsor will be assessed against the reference safety information to consider whether the event is unexpected (suspected unexpected serious adverse reaction “SUSAR”) or not. Regular clinical observations by the OAT clinic staff and physicians will secure prompt identification of potential adverse events. Any side effects or suspicious clinical conditions such as symptoms of intoxication that are observed by the clinic staff or registered using weekly questionaries, will promptly be reported to the responsible clinic physicians and the trial investigator. Emergency intoxication care and acute antidote medication (naloxone) will be available at the involved OAT clinics, and further transport to the emergency unit will be secured when close and continuous clinical monitoring is needed. Clinical and biological safety will be assessed according to the standardized scales of toxicity [34]. All unexplained grade III or IV events will lead to temporary interruption of the study medication before a new assessment is made by clinic physician and study investigators. A rapid report system for the management of SAE and SUSAR will be available.

### Frequency and plans for auditing trial conduct {23}

The decision to initiate the study will be taken in the agreement between the coordination unit and the sponsor. The study sponsor may wish to do audit visits on sites to ensure the trial is conducted according to the protocol and GCP guidelines.

### Plans for communicating important protocol amendments to relevant parties (e.g., trial participants, ethical committees) {25}

Any important protocol modifications (e.g., changes to eligibility criteria, outcomes, or analyses) will be communicated to relevant parties (e.g., investigators, ethical committees, trial participants, trial registries, journals, and regulatory organs). Any changes to the protocol will be notified to the sponsor and funder as the first action. Then, the principal investigator will notify the study centers. A copy of the revised protocol will be sent to the principal investigator to add to the Investigator Site File. Any deviations from the protocol will be fully documented using a breach report form. The protocol will be updated in the clinical trial registry.

### Dissemination plans {31a}

The findings will be presented in relevant national and international conferences and will also be presented to politicians and the health and welfare administration at all levels. The aspect in this study is considered relevant to a public audience, and several of the researchers has extensive experience with communicating research to the public through various media, as well as informing patients and other health workers. The results from the study will also be published in articles in peer-reviewed scientific journals in line with the ICMJE guidelines [35]. Open access journals indexed in PubMed/Medline will be preferred.

## Peer involvement

The project benefits a strong user involvement and close collaboration with the peer organization ProLAR (http://prolar.no), by user-representatives at the Department of Addiction Medicine, Haukeland University Hospital, Bergen. Each site will also have the possibility to involve the local user representants in the trial. The peer group is involved in the planning, design, recruitment, and implementation of the study.

## Discussion

This is the first randomized controlled trial of benzodiazepine stabilizing agonist treatment for benzodiazepine dependence. The research project will provide knowledge on the impact of such intervention on patient outcomes. We will assess efficacy and safety of stabilizing treatment with prescribed benzodiazepines compared to benzodiazepine tapering and discontinuation regarding use of illicit benzodiazepines and accordingly reducing the related risks such as overdoses due to contaminated drug supplies and improving well-being of patients with concurrent benzodiazepine and opioid dependence undergoing OAT.

Our trial involves some limitations and several strengths. For the trial, it is difficult to ensure complete blinding, however, some masking measures will be taken including blinded assessments of the study analyses by independent research staff. The study is also funded from public sources to ensure independency form pharmaceutical companies. We also have a biological primary outcome. Thus, substantial information biases are considered unlikely. The study is individually randomized, which minimizes potential confounding. The proposed sample size is powered to detect a medium effect size in between-group differences in the primary outcome. Participants will be closely monitored with weekly clinic visits and research reviews at outpatient OAT clinics where they usually are being treated and followed up. This will promote participants’ safety and retention in the study. The study protocol proposes to deliver the medication in an outpatient setting allowing participants take-home dosages to more closely mimic the service delivery situations in which the medication will be used. However, absence of a structured psychosocial intervention as a supplementary treatment, and not fully observed daily dosages are among the trial limitations. Other limitations include a non-blinded design and a possible (although low) risk of using illicitly acquired diazepam and/or oxazepam which cannot be detected by urinary analyses. However, the results of self-reported use of illicit benzodiazepines can provide additive information on the drug use patterns. One could also argue that randomization could have been stratified not only by site/city, but also by OAT medication. This, however, would have made a high number of randomization strata and many randomization blocks being incomplete with the risk of less balanced groups.

If the intervention is found to be efficacious and safe, it will be considered one of the options to standard treatment of patients with opioid and benzodiazepine co-dependency.

## Trial status

Recruiting started in September 2022. The current protocol is version 5.0 of 29-8-2024. Currently (13th of Nov 2024), we included 67 patients. Patient recruitment is estimated to be completed around December 2025.

## Data Availability

The datasets analyzed during the current study are available from the corresponding author on reasonable request.

## Abbreviations

AE: Adverse event
ANRS: Adults scaling instrument to grade the severity of adverse events
BAR: Bergen Addiction Research
BVC: Brøset violence checklist
CI: Confidence interval
CONSORT: Consolidated Standards of Reporting Trials
CRF: Case Report Files
DMC: Data Monitoring Committee
EQ-5D-5L: Euro Quality of life, 5-dimensional, 5-level questionnaire
GCP: Good Clinical Practice
ICMJE: International Committee of Medical Journal Editors
MD: Medical doctor
NORCATS: Norwegian Research Center for Agonist Treatment of Substance Use Disorder
OAT: Opioid agonist treatment
PhD: Doctor of Philosophy
ProLAR: User organization for patients receiving OAT in Norway
REC: Regional Ethical Committee
SAE: Severe adverse event
SC: Steering committee
SOP: Standard Operating Procedure
SPIRIT: Standard Protocol Items: Recommendations for Interventional Trials
VAS: Visual analog scale
SCL-10: Symptom Check List-10

## References

[1] Vold JH, Skurtveit S, Aas C, Chalabianloo F, Kloster PS, Johansson KA, Fadnes LT. Dispensations of benzodiazepines, z-hypnotics, and gabapentinoids to patients receiving opioid agonist therapy; a prospective cohort study in Norway from 2013 to 2017. BMC Health Serv Res. 2020;20(1):352. 32334602 10.1186/s12913-020-05195-5 PMC7183604

[2] Votaw VR, Geyer R, Rieselbach MM, McHugh RK. The epidemiology of benzodiazepine misuse: A systematic review. Drug Alcohol Depend. 2019;200:95–114. 31121495 10.1016/j.drugalcdep.2019.02.033 PMC6639084

[3] Abrahamsson T, Berge J, Ojehagen A, Hakansson A. Benzodiazepine, z-drug and pregabalin prescriptions and mortality among patients in opioid maintenance treatment-A nation-wide register-based open cohort study. Drug Alcohol Depend. 2017;174:58–64. 28315808 10.1016/j.drugalcdep.2017.01.013

[4] Liebrenz M, Boesch L, Stohler R, Caflisch C. Agonist substitution—a treatment alternative for high-dose benzodiazepine-dependent patients? Addiction. 2010;105(11):1870–4. 20456294 10.1111/j.1360-0443.2010.02933.x

[5] Waal H, Clausen T. Kan substitusjonsbehandling være et alternativ ved avhengighet av amfetamin/metamfetamin, kokain, cannabis og benzodiazepiner? Senter for rus-og avhengighetsorskning (SERAF). Oslo, 2020.https://www.med.uio.no/klinmed/forskning/sentre/seraf/publikasjoner/rapporter/2020/seraf-rapport-2-2020-substitusjonbehandling-andre-rusmidler.pdf.

[6] Liebrenz M, Boesch L, Stohler R, Caflisch C. Benzodiazepine dependence: when abstinence is not an option. Addiction. 2010;105(11):1877–8. 21064248 10.1111/j.1360-0443.2010.03177.x

[7] Andersson JA, Brekke M, Vallersnes OM. Akutt forgiftning ved rusrelatert bruk av benzodiazepiner. Tidsskri Nor Legeforen. 2020;140(10):1027–30. 10.4045/tidsskr.20.0035 32602327

[8] Pétursson H. The benzodiazepine withdrawal syndrome. Addiction. 1994;89(11):1455–9. 7841856 10.1111/j.1360-0443.1994.tb03743.x

[9] Petitjean S, Ladewig D, Meier CR, Amrein R, Wiesbeck GA. Benzodiazepine prescribing to the Swiss adult population: results from a national survey of community pharmacies. Int Clin Psychopharmacol. 2007;22:292–8. 17690598 10.1097/YIC.0b013e328105e0f2

[10] Norwegian Health Directorate. National guidelines for the treatment of opioid dependence. Updated May 2024. https://www.helsedirektoratet.no/retningslinjer/behandling-ved-opioidavhengighet.

[11] Aas CF, Vold JH, Skurtveitt S, Lim AG, Ruths S, Islam K, Askildsen JE, Løberg E-M, Fadnes LT, Johansson KA, INTRO-HCV Study Group. Health-related quality of life of long-term patients receiving opioid agonist therapy: a nested prospective cohort study in Norway. Subst Abuse Treat Prev Policy. 2020;15(1):68. 32883319 10.1186/s13011-020-00309-y PMC7469909

[12] Lintzeris N, Nielsen S. Benzodiazepines, methadone and buprenorphine: interactions and clinical management. Am J Addict. 2010;19(1):59–72. 20132123 10.1111/j.1521-0391.2009.00007.x

[13] Sabioni P, Bertram J, Le Foll B. Off-Label Use of Medications for Treatment of Benzodiazepine Use Disorder. Curr Pharm Des. 2015;21(23):3306–10. 26088120 10.2174/1381612821666150619092039

[14] Skeie I. Knowledge on substitution treatment with benzodiazepines in people undergoing opioid agonist treatment with co-morbid benzodiazepine dependence; 2020. [Unpublished data]. In: Norwegian Health Directorate. National guidelines for the treatment of opioid dependence. Updated May 2022. https://www.helsedirektoratet.no/retningslinjer/behandling-ved-opioidavhengighet/behandling-ved-opioidavhengighet/ved-avhengighet-til-benzodiazepiner-hos-pasienter-i-lar-anbefales-psykososial-behandling-og-nedtrapping-av-benzodiazepiner.

[15] Weizman T, Gelkopf M, Melamed Y, Adelson M, Bleich A. Treatment of Benzodiazepine Dependence in Methadone Maintenenace Treatment Patients: A Comparison of Two Therapeutic Modalities and the Role of Psychiatric Comorbidity. Aust N Z J Psychiatry. 2003;37(4):458–63. 12873331 10.1046/j.1440-1614.2003.01211.x

[16] Barker MJ, Greenwood KM, Jackson M, Crowe SF. Cognitive effects of long-term benzodiazepine use: a meta-analysis. CNS Drugs. 2004;18(1):37–48. 14731058 10.2165/00023210-200418010-00004

[17] Park TW, Larochelle MR, Saitz R, Wang N, Bernson D, Walley AY. Associations between prescribed benzodiazepines, overdose death and buprenorphine discontinuation among people receiving buprenorphine. Addiction. 2020;115(5):924–32. 31916306 10.1111/add.14886 PMC7156323

[18] Macleod J, Steer C, Tilling K, Cornish R, Marsden J, Millar T, Hickman M. Prescription of benzodiazepines, z-drugs, and gabapentinoids and mortality risk in people receiving opioid agonist treatment: Observational study based on the UK Clinical Practice Research Datalink and Office for National Statistics death records. PLoS Med. 2019;16(11):e1002965. 31770388 10.1371/journal.pmed.1002965 PMC6879111

[19] Edvardsen HME, Clausen T. Opioids related deaths during 2000–2017 in Oslo. Oslo University Hospital; 2020. https://oslo-universitetssykehus.no/fag-og-forskning/nasjonale-og-regionale-tjenester/rettsmedisinske-fag/alkohol-og-r.

[20] Holland A, Copeland CS, Shorter GW, Connolly DJ, Wiseman A, Mooney J, Kevin Fenton K, Harris M. Nitazenes-heralding a second wave for the UK drug-related death crisis? Lancet Public Health. 2024;9(2):e71–2. 10.1016/S2468-2667(24)00001-X. (Epub 2024 Jan 12). 38224702 10.1016/S2468-2667(24)00001-X PMC7617954

[21] Maust DT, Petzold K, Strominger J, Kim HM, Bohnert ASB. Discontinuation and Mortality Among Patients With Long-Term Benzodiazepine Therapy. JAMA Netw Open. 2023;6(12): e2348557. 10.1001/jamanetworkopen.2023.48557. 38117495 10.1001/jamanetworkopen.2023.48557 PMC10733804

[22] Voshaar RC, Gorgels WJ, Mol AJ, van Balkom AJ, Mulder J, van de Lisdonk EH, et al. Predictors of long-term benzodiazepine abstinence in participants of a randomized controlled benzodiazepine withdrawal program. Can J Psychiatry. 2006;51:445–52. 16838826 10.1177/070674370605100706

[23] SPIRIT (Standard Protocol Items: Recommendations for Interventional Trials). 2013. http://www.spirit-statement.org/. 10.1590/2177-6709.27.3.e2220290.oar PMC9255961 35792787

[24] Derogatis LR, Lipman RS, Rickels K, Uhlenhuth EH, Covi L. The Hopkins Symptom Checklist (HSCL): a self-report symptom inventory. Behav Sci. 1974;19(1):1–15. 4808738 10.1002/bs.3830190102

[25] Herdman M, Gudex C, Lloyd A, Janssen M, Kind P, Parkin D, Bonsel G, Badia X. Development and preliminary testing of the new five-level version of EQ-5D (EQ-5D-5L). Qual Life Res. 2011;20(10):1727–36. 21479777 10.1007/s11136-011-9903-x PMC3220807

[26] Reaction time test (online version). How old are your reactions? https://www.justpark.com/creative/reaction-time-test/. Accessed 15 Aug 2022.

[27] Almvik R, Woods P, Rasmussen K. The Brøset Violence Checklist: Sensitivity, specificity and interrater reliability. J Interpers Violence. 2000;15:1284–96.

[28] Rosner B. Fundamentals of Biostatistics. 7th ed. Boston, MA: Brooks/Cole; 2011.

[29] Moher D, Schulz KF, Altman DG. The CONSORT statement: revised recommendations for improving the quality of reports of parallel-group randomised trials. Lancet. 2001;357(9263):1191–4. 11323066

[30] DAMOCLES Study Group. A proposed charter for clinical trial data monitoring committees: helping them to do their job well. Lancet. 2005;365(9460):711–22. 15721478 10.1016/S0140-6736(05)17965-3

[31] Ramsey SD, Willke RJ, Glick H, Reed SD, Augustovski F, Jonsson B, Briggs A, Sullivan SD. Cost-Effectiveness Analysis Alongside Clinical Trials II—An ISPOR Good Research Practices Task Force Report. Value Health. 2015;18(2):161–72. 25773551 10.1016/j.jval.2015.02.001

[32] Oksazepam (Sobril). Summary of Product Characteristics (SPC). Norwegian Medical Products Agency. Updated August 2021. https://www.legemiddelsok.no/sider/default.aspx?searchquery=Valium&f=Han;MtI;Vir;ATC;Var;Mar;Mid;Avr;gen;par;&pane=0.

[33] Diazepam (Valium). Summary of Product Characteristics (SPC). Norwegian Medical Products Agency. Updated January 2021. https://www.legemiddelsok.no/sider/default.aspx?searchquery=Valium&f=Han;MtI;Vir;ATC;Var;Mar;Mid;Avr;gen;par;&pane=0.

[34] ANRS scale to grade the severity of adverse events in adults. 2008. http://www.anrs.fr/content/download/2242/12805/file/ANRS-GradeEI-V1-En-2008.pdf.

[35] International committee of medical journal editors. Updated January 2024. http://www.icmje.org/recommendations/. 10.12771/emj.2024.e48 PMC12093628 40704003

